# A lightweight YOLO11n seg framework for real time surface crack detection with segmentation

**DOI:** 10.1038/s41598-026-37073-1

**Published:** 2026-01-29

**Authors:** Shweta Tiwari, Kamal Kumar Gola, Rohit Kanauzia, Gopal Kumar Gupta

**Affiliations:** 1 Department of Computer Science and Engineering, Chandigarh University, Mohali, India; 2 Department of Computer Science and Engineering, Graphic Era Deemed to be University, Dehradun, India; 3 Department of Computer Science and Engineering, Swami Rama Himalayan University, Dehradun, India; 4 Symbiosis Institute of Technology Nagpur Campus, Symbiosis International (Deemed University), Pune, India

**Keywords:** Superficial cracking recognition, YOLO, Deep Learning, Lexical segmentation, Structural health monitoring, Engineering, Mathematics and computing

## Abstract

The recognition of superficial cracks is essential to ensure the safety, durability, and longevity of civil infrastructure such as bridges, pavements, tunnels, and buildings. Traditional crack detection methods have been largely based on manual inspections and classical image processing techniques, including edge detection, thresholding, and morphological operations. With the rapid advancement of computer vision and deep learning, significant progress has been made in automating crack detection. To gain insight into previous research, we reviewed some studies from the past few years and identified YOLO11 as the most suitable model for crack detection tasks. In this study, we propose a deep learning-based framework for surface crack detection using the Crack-Seg dataset and the YOLO11n-seg architecture. Experimental results demonstrate that YOLO11n-seg achieves strong performance on the Crack-Seg dataset. The suggested model reaches a Precision of 78.8%, which is comparable to heavy baselines. Our results show that the suggested lightweight model, with just 2.8 million parameters, has a Box mAP@50 of 76.2% with a Mask mAP@50 of 58.7%. Most importantly, the model reaches an inference rate of 3.6ms for each image (on Tesla T4), allowing for ultra-fast processing in highly automated inspection systems. These findings establish a new benchmark for edge-deployable crack recognition, demonstrating the possibility that the YOLO11n-seg architecture may provide acceptable segmentation performance with lower computational cost than large, traditional methods.

## Introduction

Modern societies rely on civil infrastructure, including highways, bridges, underground walkways, dams, and buildings. The security, longevity and accessibility of these building components are critical, as failures can result in not only expensive restorations, but also catastrophic events and loss of life. Surface cracks are one of the first symptoms of structural deterioration and are commonly caused by strain, environmental factors, exhaustion, or material degradation. The early and precise identification of these cracks is critical for preventive maintenance, condition surveillance, and effective allocation of resources^11^. Traditionally, crack inspection relied mainly on manual surveys conducted by qualified engineers. These manual procedures, while accurate in isolated circumstances, are expensive, time-consuming, subjective, and unsuitable for massive infrastructure networks. As a result, the demand for automatic, durable, and economical crack detection technologies has increased dramatically in recent years. During the last two decades, researchers have investigated a variety of computer algorithms for the identification of fractures^30^. A researcher designed a classification model specially detecting the cracks of road^1^. A review study collects and analyzes widely used crack datasets for classification, object identification, and segmentation applications. It also investigates annotation techniques, metrics for evaluating loss functions, and representational deep learning architectures. The review identifies important issues at the data, design, and system levels, and it suggests engineering-oriented evaluation criteria^29^. Early attempts relied on traditional image processing approaches such as edge detection, adaptive filtering, and morphological procedures. Although these technologies worked well in controlled contexts, they suffered in real-world scenarios due to varied surface textures, lighting adjustments, reflections, and environmental noise^3^. To solve these problems, deep learning and machine learning techniques were established. To characterize fractures, traditional machine learning techniques like Support Vector Machines (SVMs) and Random Forests used handmade features such as Histogram of Oriented Gradients (HOG), Local Binary Patterns (LBP), and Gabor filters. However, the heterogeneity in the appearance of the fractures hindered the generalizability of these models^7^. The emergence of deep learning, especially Convolutional Neural Networks (CNNs) and revolutionized computer vision opened up new avenues for crack detection. Models like AlexNet, VGG, and ResNet demonstrated promising results for image classification applications, and their concepts were applied to crack detection^2^. subsequently developed fully convolutional networks such as U-Net and Mask R-CNN improved the problem by allowing pixel-level crack segmentation^34^.While these models provided excellent precision with accurate crack recognition, they came with a high computational cost, substantial memory usage, and poor inference speeds. These constraints limit their usefulness for immediate crack monitoring, particularly in mobile robotic devices, drones, and edge sensors with limited resources^26^. In recent times, object detection algorithms have already been integrated into crack recognition pipelines to address some of the constraints mentioned above. The You Only Look Once (YOLO) series of algorithms has received a lot of attention because of its ability to reconcile precise detection with real-time performance. YOLO models have been optimized to identify objects in a single forward pass, therefore being significantly more efficient than region proposal-based approaches like Faster R-CNN.With successive versions (YOLOv3, YOLOv5, YOLOv8 and the most recent variants of YOLO11), these algorithms feature architectural enhancements, mechanisms of attention, and segmentation heads, broadening their application to tasks such as object segmentation, surveillance, and anomaly detection^20^.

Given this background, the current research explores the subsequent research questions.

- RQ1: How accurate is YOLO11n-seg in detecting or segmenting surface cracks when compared to conventional image processing and deep learning techniques?
- RQ2: How efficient is YOLO11n-seg in minimizing false positives along with false negatives compared to conventional crack detection methods?
- RQ3: Is YOLO11n-seg appropriate for immediate application with structural health monitoring systems, with acceptable accuracy-speed trade-offs?

The subsequent sections explains the purpose and details of the experimental work. Section "Development of deep learning algorithms for superficial crackingRecognition" explains the development of DL algo followed by a comparative literature review. The methodology and the evaluation parameters are represented in section “Methodology”. Finally, the observation and discussion section explains the results of the article.

## Development of deep learning algorithms for superficial cracking recognition

**Fig. 1:**
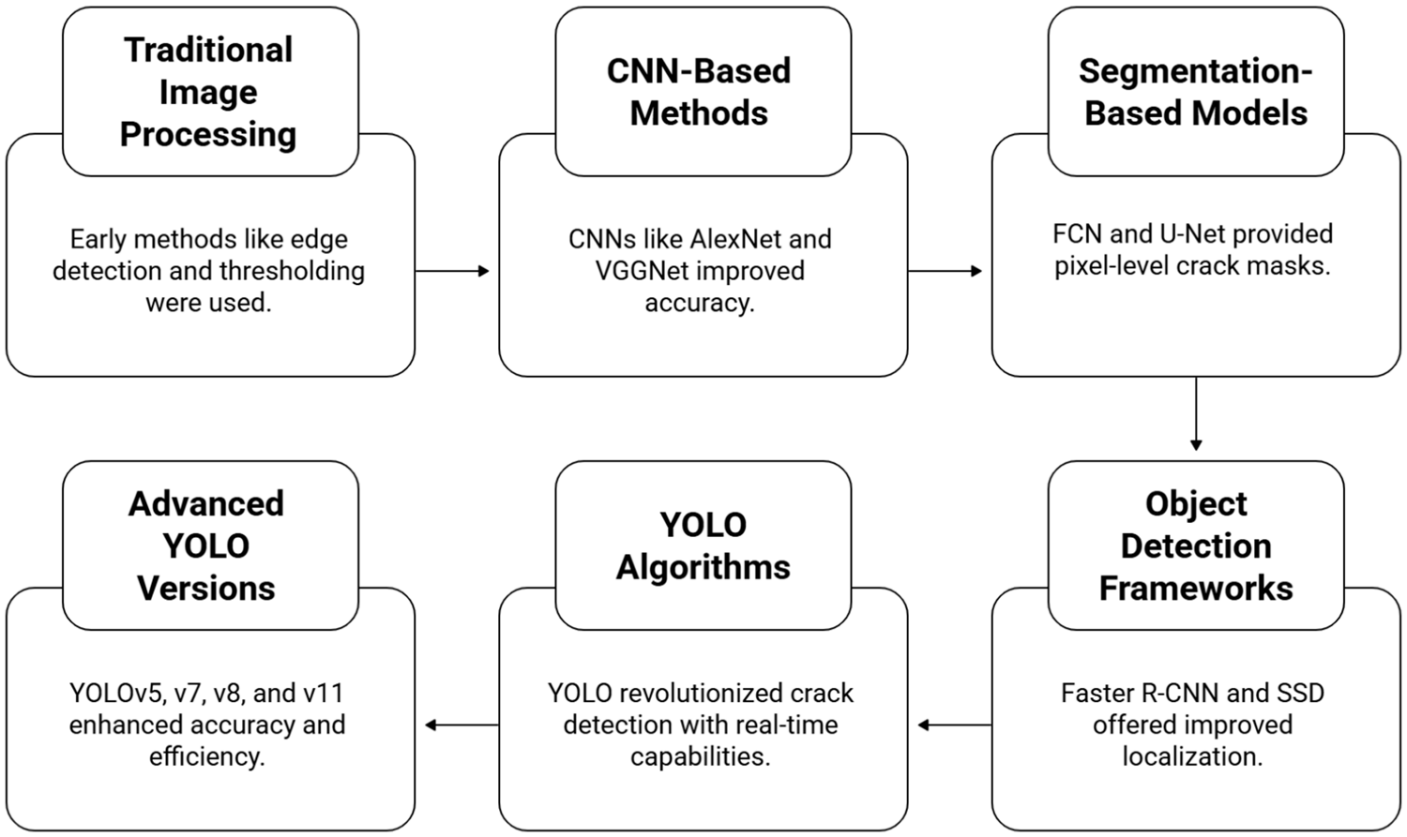
Evolution of deep learning models for surface crack detection: from CNN classifiers to segmentation networks, Faster/Mask R-CNN, and YOLO-based detectors.

Surface fracture detection in building structures has gone beyond conventional image processing to powerful deep learning algorithms. Early solutions, including edge detection and thresholding, were computationally straightforward but unreliable in the presence of noise, low lighting, and irregular fracture patterns^14^. The emergence of convolutional neural networks (CNNs) heralded a significant shift, as designs such as AlexNet, VGG, and ResNet could extract hierarchical characteristics for crack categorization. Pixel-based CNNs increased accuracy, but lacked exact localization, resulting in coarse output^17^. To cope with this, segmentation models like Fully Convolutional Networks (FCN) along with U-Net enabled pixel-level crack identification, resulting in detailed crack morphology^23^. U-Net gained popularity because of its encoder-decoder architecture with skip connections, but computational costs hindered real-time applications. Object detection frameworks were developed subsequently, with faster R-CNN producing correct region suggestions, SSD providing quicker estimation, and Mask R-CNN integrating detection and segmentation^15^. However, the trade-offs between speed and robustness persisted. YOLO series reframed crack detection as a single regression task, allowing the class prediction for the concurrent bounding box^37^.. YOLOv3 exhibited real-time practicality, although successive versions such as YOLOv7, YOLOv8, and YOLOv11, included advances such as feature pyramid network, path aggregation, and attention modules, which considerably improved accuracy and efficiency^21^. The lightweight YOLO variations made it easier to deploy using drones and smartphones, while YOLOv8-Seg expanded the capabilities to include real-time segmentation^9^. The transition from CNN classifiers to current YOLO detectors underscores the quest for precision and effectiveness, with YOLO emerging as the leading method for adaptable, immediate crack detection in intelligent infrastructure monitoring. The evolution of deep learning models for surface crack detection from traditional image processing models to the latest YOLO versions are shown in Fig. 1. This describes the details from CNN classifiers to segmentation networks, Faster/Mask R-CNN, and YOLO-based detectors.A comparative analysis of YOLO-based Surface Crack Detection is shown in Table 1 while Table 2 shows the enhanced features of YOLO11 facilitating Surface Crack Detection.

## Literature review

Surface crack detection is a vital component of construction and repairs, encompassing roads, bridges, pavements, including structural buildings. Traditional image processing approaches (edge detection, morphological functions) had inadequate robustness when dealing with changeable lighting, complex backdrops, and fine crack patterns, as shown in Fig. 1. Convolutional neural networks (CNNs) made automatic feature extraction possible, which improved detection accuracy^22^^37^. However, early CNN-based approaches were limited to categorization or patch-level detection, with no exact localization or real-time capacity. YOLO (You Only Look Once) object recognition systems have improved crack detection research by detecting cracks in real time with great accuracy. The following overview brings together major theoretical advancements and trends from YOLO-based crack detection investigations.

### The evolution of the YOLO

#### The real-time applications of early versions of YOLO

A researcher used YOLOv3 to detect cracks on pavements and achieved good accuracy (88%) by real-time inference^22^. Similarly, in some other work researcher modified YOLOv3 enabling bridge crack detection, making backbone changes to increase localization^37^. A research used YOLOv3 with UAVs in real-time building inspections, obtaining a high FPS while experiencing some precision loss for fine boundaries^4^. These investigations show that while YOLOv3 allows for realistic real-time deployment, it falls short of two-stage detectors in terms of fine-grained boundary precision.

#### Lightweight and edge-oriented versions of YOLO

YOLOv5 established lightweight architectures that are ideal for UAV and mobile deployments. A framework which designed GC-YOLOv5s that enable UAV-based road inspection, focusing on edge device efficiency while retaining competitive mAP^31^. Another framework used YOLOv5 and Bottleneck Transformers (YOLOv5-CBoT) to recognize long-span cracks and contextual characteristics^33^. While some author incorporated attention techniques into YOLOv5 to raise its resilience to complicated environments^24^. These enhancements emphasize the equilibrium of model size, inference speed, and detection accuracy, allowing realistic deployment using UAVs and mobile devices.

#### Sophisticated task-specific models

Recent research has focused on YOLOv8 to YOLOv11 providing high-performance task-specific fracture detection. Han (2024) used MS-YOLOv8 in multiscale pavement detection^12^. Some authors enhanced YOLOv8 for structural fracture identification, resulting in higher mAP and FPS^35^^18^. A few proposed BCCD-YOLO on exposed concrete surfaces, claiming a 3.3% improvement in precision over YOLOv8^25^. Some presented YOLO11-BD in bridge inspection, which combines lightweight design and low latency^8^. While one demonstrated Flexi-YOLO, which improves mAP@0.5 but preserves high FPS in complicated pavement environments^32^. These studies represent a trend towards unique, lightweight architectures that retain real-time capabilities while including segmentation or attention-based enhancements.

**Table 1:** Enhanced features of YOLO11 facilitating Surface Crack Detection.

Author & Year	Feature Area	Problem with Earlier YOLO Implementation	Enhanced Feature in YOLO11	YOLO11 Improvement (Surface Crack Detection Context)
G. Yu, 2023^24^	Pavement Crack Detection	Could not capture long-range dependencies; failed on elongated or fragmented cracks	Lightweight + Bottleneck Transformer (long-range context)	YOLOv5-CBoT with Bottleneck Transformer integration
T. Xu, 2025^14^	Small Object Detection	Struggled with detecting very small cracks due to limited feature fusion and weak attention on fine details	Enhanced SPP/SPPF + attention (better small-object detail)	Slim-YOLO with MDFFAM and LKSPP modules
X. Zheng, 2023^22^	Remote Sensing Detection	Low precision in detecting small cracks in complex backgrounds; insufficient multi-scale context	Feature-enhanced multi-scale fusion and spatial-context guidance	ESL-YOLO with feature enhancement and spatial-context-guided fusion network
Y. Zhang, 2020^28^	Road Crack Detection	Limited multi-scale detection; poor performance on UAV images with varying crack sizes	Edge/device optimizations (efficient blocks, multi-scale, pruning)	USSC-YOLO with enhanced multi-scale detection for UAV images
W. Ren, 2025^19^	Concrete Crack Detection	Missed fine cracks; lacked focused attention on crack regions; low recall	Attention-driven neck: channel and spatial attention modules	BCCD-YOLO with enhanced feature fusion and attention mechanisms

The conceptual progression of YOLO-based crack detection extends through generic YOLOv3 systems to lightweight, task-specific, immediate models (YOLOv5-YOLOv11). The major contributions include high frame rates with UAV along with edge deployment. The lightweight and attention-augmented variations of the YOLO frameworks improved the efficiency and robustness of the system. YOLO models with segmentation capabilities approximate Mask R-CNN boundary resolution. Table 1 summarizes the enhanced features of YOLO11 that facilitate Surface Crack Detection.

### Experimental elements for YOLO-inspired crack detection

#### Concurrent installation

The primary goal of an experimental setup is to provide immediate crack identification using UAVs, edge devices, including mobile platforms. YOLO-based approaches, such as MobileYOLO along with YOLO11-BD, provide significant efficiency benefits while maintaining detection accuracy^8^.

####  Accuracy vs framework complexity

Maintaining the balance between accuracy and computing efficiency is critical for prediction systems. Two-stage detectors, such as Faster R-CNN and Mask R-CNN, excel at boundary-level precision but are computationally intensive^37^^4^. YOLO-based models offer excellent detection accuracy with a short inference time, while lightweight variations improve edge/mobile installation feasibility^31^^33^.

####  Task-dependent adaptations

YOLO designs are increasingly adapted for deployment situations.

- Pavement assessment: Flexi-YOLO^32^, YOLOv5-CBoT^33^.
- Bridge inspections: YOLO11-BD^8^; SPF-YOLOv11^36^^25^ refer to bare concrete surfaces as BCCD-YOLO.
- UAV/mobile evaluation: GC-YOLOv5s; MobileYOLO^31^.

These modifications rely heavily on multiscale feature integration, attention segments, and edge optimization.

#### Constraints and research shortcomings

Despite the excellent progress of yolo, there are also some difficulties. According to some researchers, YOLO-based models still fall behind Mask R-CNN in terms of boundary precision for fine fractures^4^^37^. Model performance may suffer under different surface textures and lighting circumstances due to dataset specificity^12^^31^. Lightweight models may not detect low-contrast and hairline cracks^33^^25^. The reason behind this is that the premium, annotated crack databases are limited, hampering training and evaluation. A comparative analysis of some of the previous research under the domain of Surface Crack Detection based on the YOLO algorithm is shown in Table 2.

### Analysis of architecture: YOLO8 Vs YOLO11

YOLOv8 has proven to be a reliable baseline, but in present study we choose YOLO11 architecture due to unique structural changes that promote the identification of highly frequent, thin-bodied features that include concrete cracks. To enhance feature extraction,YOLOv8 makes use of the C2f (Cross-Stage Partial with 2 branching) module, which divides the input feature map X into two sections to enhance gradient flow. YOLO11 features the C3k2 block, an improved bottleneck structure. Unlike the usual C2f, C3k2 uses an adaptive kernel selection technique that adjusts the receptive field in response to feature variance.

Let the input feature map be denoted as $X \in \mathbb {R}^{C \times H \times W}$. The output feature map *Y* of the C3k2 block is computed as:

1 $$\begin{aligned} Y = \text {Concat}\left( \Psi \left( X_{\text {split1}}\right) , \; \Phi \left( X_{\text {split2}}, \text {Kernel}_{\text {dynamic}}\right) \right) \end{aligned}$$

Here, $\Psi (\cdot )$ denotes the lightweight convolutional path, while $\Phi (\cdot )$ represents the adaptive convolutional branch with dynamically selected kernel sizes $k \in \{3 \times 3,\; 5 \times 5\}$, determined by the local texture density. Surface cracks can be represented as quasi-one-dimensional topologies residing within a two-dimensional space from the standpoint of crack identification. The network can simultaneously record narrow crack widths using small kernels ($3 \times 3$) and maintain long-range crack continuity using bigger kernels ($5 \times 5$) thanks to the variable receptive field in C3k2. The fractured segmentation artifacts that are frequently observed in YOLOv8-based models are much reduced by this technique.

#### Noise suppression using spatial attention

YOLOv8 is sensitive to ambient noise (oil stains, rough surfaces) during concrete assessment. YOLO11 overcomes this problem by replacing traditional constraints with the C2PSA (Cross-Stage Partial with Spatial Attention) module. The C2PSA module includes a spatial attention map $A_s$, which is used to recalibrate the significance of features geographically.

The spatial attention weight is obtained as follows using an intermediate feature map *F*:

2 $$\begin{aligned} A_s(F) = \sigma \left( \text {Conv}{1\times 1}\left( \text {ReLU}\left( \text {Conv}{1\times 1}\left( \text {AvgPool}(F) \right) \right) \right) \right) \end{aligned}$$

The refined feature output $F'$ is then obtained by:

3 $$\begin{aligned} F' = F \otimes A_s(F) \end{aligned}$$

where $\sigma (\cdot )$ denotes the Sigmoid activation function and $\otimes$ represents element-wise multiplication.

Convolutional feature responses can be dominated by the prominent and uneven visual patterns that backdrop concrete textures frequently display in the context of surface crack analysis. By suppressing these background activations and highlighting elongate and high-contrast crack locations, C2PSA’s spatial attention mechanism improves segmentation accuracy and resistance to noise.

Recent research on pavement crack detection shows a clear and connected evolution toward efficient, accurate, and practically deployable deep learning solutions. Lightweight encoder–decoder networks were introduced to address the challenges of complex backgrounds and limited computational resources by integrating residual learning, attention mechanisms, and efficient convolutions to enhance the representation of characteristics while reducing model complexity^38^. Building on this foundation, automated network design frameworks further reduced human intervention by employing optimization algorithms to automatically discover compact yet effective architectures, enabling scalable and efficient crack detection systems^39^. To better handle cracks of varying widths and subtle details, multi-frequency learning strategies were proposed, explicitly separating high- and low-frequency features to preserve fine crack structures while maintaining strong global representations^19^. In parallel, ensemble learning approaches improved robustness by combining multiple neural networks, mitigating the weaknesses of individual models and enabling more reliable crack detection and measurement^10^. More recently, closed-loop feedback frameworks that incorporate adversarial learning addressed the limitations of open-loop segmentation models by enabling automatic error correction and improved continuity of crack detection^16^. Together, these works reflect a systematic progression towards intelligent, lightweight, and adaptive pavement crack detection frameworks capable of operating effectively in real-world inspection scenarios.

**Table 2:** Comparative Study of YOLO-based Surface Crack Detection (2019–2025).

Author(s) & Year	Algorithm & Focus	Objectives	Deployment Area	Model Performance	Strengths	Limitations
Nie & Wang, 2019^22^,	YOLOv3—pavement crack detection	Demonstrate YOLOv3 for pavement crack detection and evaluate accuracy/speed	Pavement/road images	$\sim$88% detection accuracy	Real-time capability; simple pipeline	Limited pixel-level segmentation; moderate small-crack recall
Zhang, 2020,^37^	Improved YOLOv3 for bridge crack detection	Improve YOLOv3 backbone/PANet for better localization on bridge surfaces	Bridge surfaces	Higher mAP than baseline YOLOv3	Better localization; tailored augmentations	Limited boundary segmentation vs Mask R-CNN
Babu, 2023^4^,	YOLOv3 + UAV pipeline	Enable on-board/real-time UAV building inspections	UAV (building)	Real-time FPS on 1080p	Practical UAV integration; real-time inference	Slightly lower precision for boundaries
Xiang, 2023,^31^	YOLOv5s + GC module	Reduce compute, retain accuracy for UAV deployment	UAV road inspection	Improved FPS and competitive mAP	Lightweight; suitable for edge/UAV	May miss fine hairline cracks; dataset-specific tuning
Yu, 2023^33^,	YOLOv5 + Bottleneck Transformer	Capture long-range dependencies for long-span cracks	Pavement/road imagery	Higher precision/F1 vs baseline YOLOv5	Handles long continuous cracks	Higher compute than vanilla YOLOv5
Ren, 2022,^24^	YOLOv5 + attention	Improve detection in complex backgrounds	Pavement inspection	Better precision/recall than baselines	Robust to varying backgrounds	Not pixel-perfect segmentation
Jiang et al., 2023,^13^	Edge-optimized YOLOv5	Suitability for low memory/compute devices	Edge/mobile systems	Good FPS on embedded HW	Edge-optimized; low latency	Reduced capacity for subtle cracks
Han, 2024,^12^	MS-YOLOv8	Enhance multi-scale/complex target detection	Pavement/road	Higher mAP vs YOLOv8	Strong multi-scale detection	Requires careful tuning; heavier than nano variants
Zhang, 2025,^35^	Optimized YOLOv8	Structural crack detection accuracy and speed	Structural concrete (UAV/handheld)	mAP gains, stable FPS	Optimized for structural textures; good recall	Inferior to segmentation for boundaries
ACM Author, 2025^18^,	YOLOv8 adaptation for concrete	Accurate crack detection leveraging YOLOv8	Concrete/pavement	Improved detection metrics	Fast and accurate	Limited to box/instance granularity
Ren, 2025,^25^	BCCD-YOLO	Boost precision on bare concrete	Bare concrete surfaces	+3.3% precision over YOLOv8	High precision for bare concrete	May not generalize across textured pavements
Dong, 2025,^8^	YOLO11-BD (lightweight)	Bridge crack detection with nano/lightweight design	Bridge inspections (embedded/UAV)	Higher accuracy; lower latency	Lightweight; low inference latency	Limited external validation so far
R. Zhang, 2025,^36^	SPF-YOLOv11	Fine-crack detection in bridges	Bridge concrete	Large precision/recall/mAP improvements	Fine-detail retention; high mAP	Needs high-res input and tuned preprocessing
Yang, 2025,^32^	Flexi-YOLO (YOLOv8 variant)	Robust/efficient detection with high FPS	Pavement (mobile/UAV)	+1.5% mAP@0.5; higher FPS	Lightweight; robust to complex backgrounds	Requires cross-dataset testing
Xiang et al., 2023,^31^	YOLOv5s variants	Improve UAV inference/detection with lightweight modules	UAV-based road inspection	Competitive mAP; favorable FPS	Edge-suitable; practical UAV use	Dataset-specific retraining needed

### Combining segmentation with real-time performance

The object detection(bonding box) model has problems in addressing the noise and quantifying the severity of damage, they also have speed limitations. There is a clear contrast between high-speed detection frameworks and computationally demanding segmentation models, according to recent research. The comparison of segmentation and real time detection approaches is clearly explained in Table 3.

#### Methods of high-fidelity segmentation

Some of the research put segmentation quality ahead as compared to speed. Diffusion probabilistic models (CrackDiffNet) designed to produce robust fracture identification with high Intersection with Union (IoU) scores^28^. However, this method is less appropriate for real-time deployment due to its significant processing costs. In a similar vein, a researcher exploited multiscale features to produce superior border delineation while concentrating on fine-grained identification for UAV bridge inspections. The technique has significant memory consumption despite its high resolution (HR) precision, which presents problems for edge devices with limited resources^5^. Although particular speed metrics were not emphasized as a key strength, a study (UCSDC) concentrates on an autonomous pixel-level algorithm, to enhance the pavement evaluation and noise reduction^27^.

#### Real-time detection methods

On the other hand, a research used YOLOv5 to optimize the inference efficiency for road inspections. Although this method effectively produced real-time performance, which was appropriate for high-speed assessment. But it lacks pixel-level segmentation, offering only bounding box identification, which is inadequate for in-depth structural width analysis^6^.

#### The transition to lightweight segmentation

In an effort to minimize the gap, more recent research (such as the 2025 Lightweight study) suggests lightweight architectures made especially for real-time road surveillance. By directly addressing the shortcomings of previous heavy segmentation models and rough detection-only models, these techniques seek to provide rapid and accurate performances with optimized computational loads^39^.

**Table 3:** Summary of related work comparing segmentation and real time detection approaches for crack analysis.

Author(s) & Year	Algorithm & Focus	Objectives	Deployment Area	Model Performance	Strengths	Limitations
Wang et al. (UCSDC)^27^	Self-calibrated CNN	Improve pixel-level boundary accuracy	Pavement and bridge Assessment	Improved Precision and Recall	Noise reduction	Limited curved crack identification
Song et al. (2025)^28^	Diffusion Probabilistic Models	Robust crack segmentation	Structural Health Monitoring	High IoU	Noise robustness	High computational cost
Chu & Chun. (2024)^5^	Multiscale cascaded CNN	Fine-grained segmentation in HR images	UAV Bridge Inspection	High HR Accuracy	Excellent boundary	High memory usage
Guijie Zhu (2025)^39^	Lightweight CNN DPSO	Real time crack detection	Road Monitoring	Fast & Accurate	Optimized Architecture	Complex training
Dong et al. (2021)^6^	YOLOv5 Object Detection	Real time crack detection	Road Inspection	High Speed	Real-time capable	No pixel segmentation

## Methodology

**Fig. 2:**
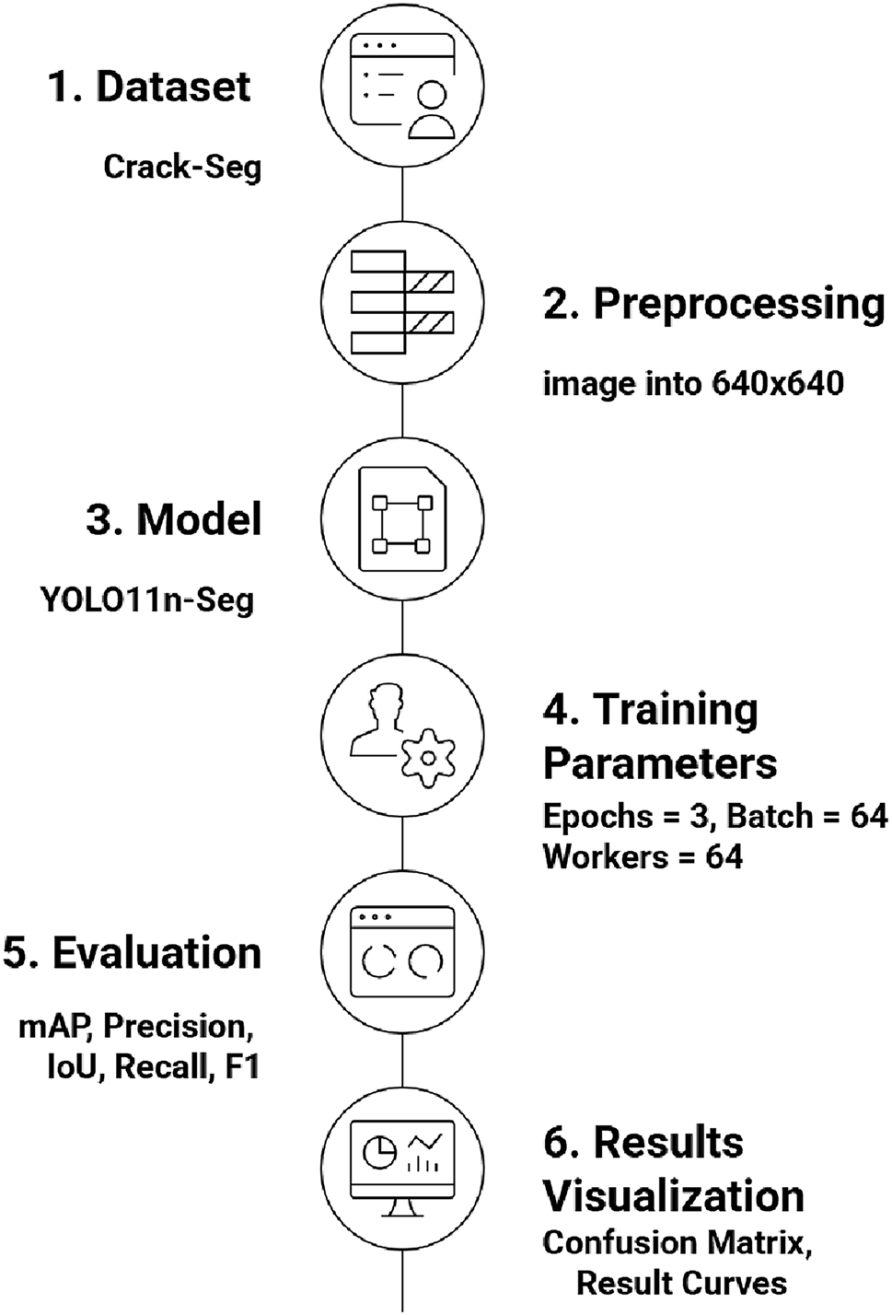
Methodology specification and control flow.

Superficial crack recognition in concrete structures requires a reliable technology that can capture tiny crack details while being efficient for real-time deployment. In this experiment, we use the YOLO11n-Seg model, a lightweight and powerful form of the YOLO (You Only Look Once) algorithm that is specifically designed for segmentation tasks, as shown in Fig. 2. In structural health monitoring (SHM), simply localizing a defect is insufficient; the severity of the damage is determined by its physical dimensions (width, length, and area). Despite classification or bounding box approaches, segmentation-oriented YOLO allows both fracture location and contour delineation, which is critical for structural integrity investigation. The methodology streamlines data set collection and preparation, model construction, training, and validation into a single procedure, enabling consistency and adaptability for real-world inspection.

### The dataset details

This study used the Crack-Seg dataset, which was initially developed by Zhang et al. (2016) at Tongji University. This dataset provides more than 11,000 crack and non-crack images created using 118 high-resolution road photographs. It delivers pixels-level annotated road surface crack photos, allowing supervised training for crack detection and segmentation tasks. (Data Split:) To guarantee rigorous examination, the dataset was split into two sets: training (80%) and validation (20%). (Annotation Format:)We used the YOLO default segmentation format (polygon coordinates standardized to [0,1]). To ensure interoperability within the YOLO11n-Seg input layer, the dataset was subjected to preprocessing processes that included resizing images to 640 $\times$ 640 pixels. Crack-seg.yaml describes the dataset’s configuration, including the path for validation and training data, class labels, and preprocessing parameters.

### The model architecture

The suggested detection methodology proposed in this research is based on YOLO11n-Seg which uses the yolo11n-seg.pt pre-trained weights file. This model, a segmentation-capable micro version of YOLO11, provides a great balance of computing efficiency and precision. To achieve robust performance for the model, we used the YOLO11n-seg architecture. The training was performed on a Tesla T4 GPU with the following hyperparameters: Optimizer: SGD with velocity of 0.937, initial learning rate is lr=0.01 with a cosine decay schedule, image input size was 640 $\times$ 640, batch size and worker details were 64. The AdamW optimizer, using the autonomous learning rate scheduling, is used to iteratively update the model weights. The training stopped when performance measures (mAP50) reached a plateau, suggesting convergence. The model displayed quick parameter adaptation caused by the effectiveness of transfer learning. Epoch 9 had significant performance gains, with Precision increasing to 78.8% and Mask mAP50 reaching 58.7%. The training & prediction workflow is directly mapped to the progressive algorithmic structure of the methodology. The developed YOLO11 Surface Crack Segmentation model is then used for inference, producing segmentation masks and bounding boxes that draw attention to surface fractures. Algorithm 1 provides a summary of the entire methodology.

**Algorithm 1:**
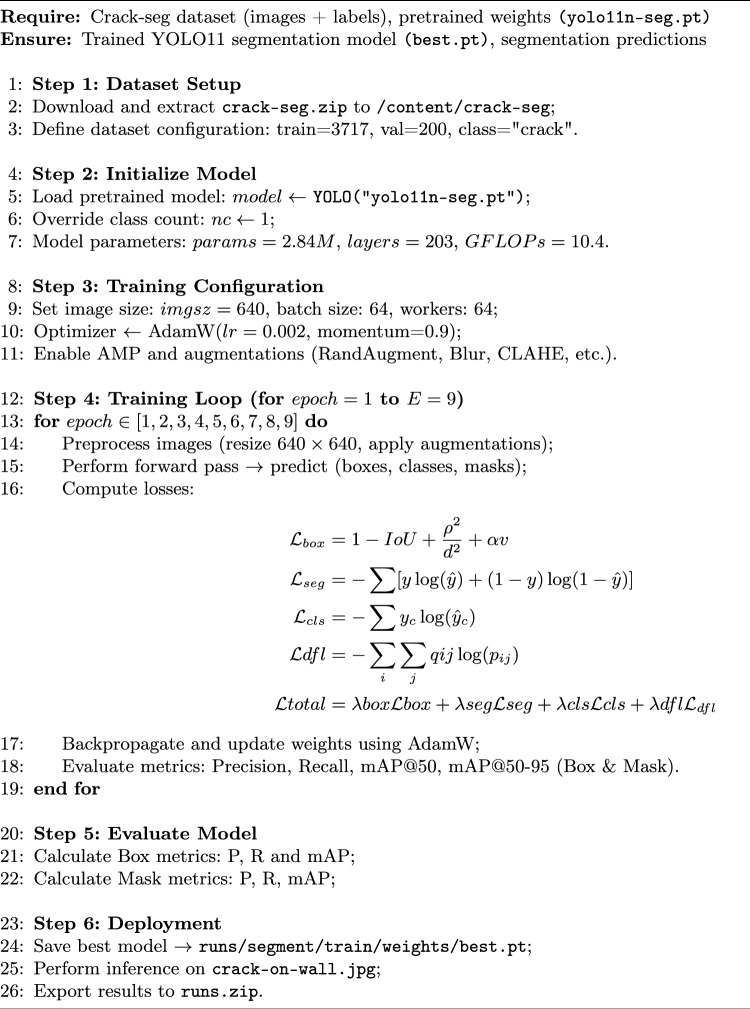
YOLO11_Surface_Crack_Segmentation

### Experimental setup

**Fig. 3:**
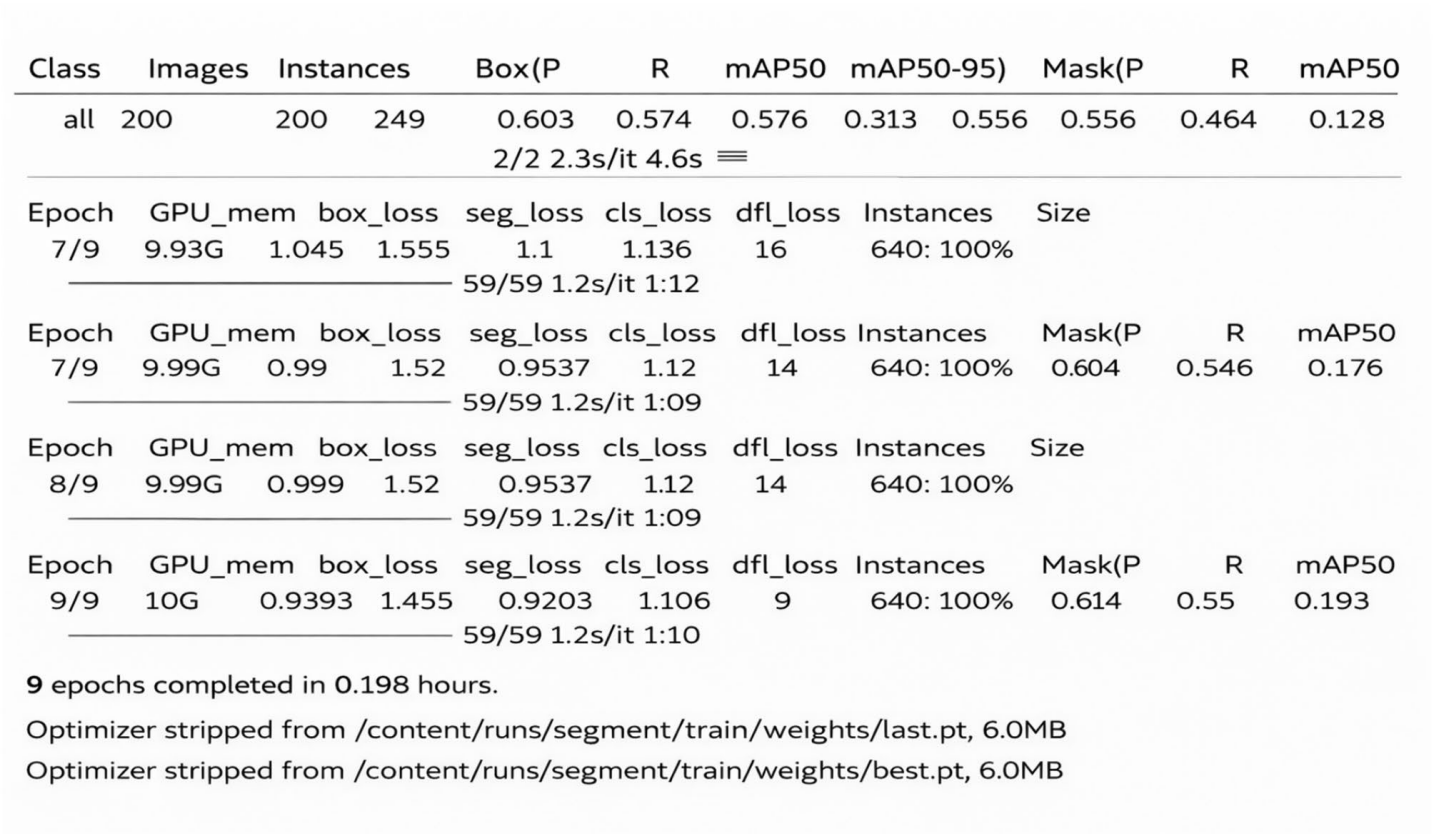
Training specifications of the developed model.

The developed Ultralytics YOLO11 Surface Crack Segmentation model was used for training and evaluation studies. The model was then executed for inference, producing segmentation masks and bounding boxes that draw attention to surface fractures. Algorithm 1 provides a summary of the entire methodology. Training was performed in a GPU-enabled environment, with parallel workers used to optimize data set loading. The training parameters were 9 epoc, batch 64, and worker 64,these details are shown in Fig. 3. During training, a part of the Crack-Seg dataset was set aside for validation to track performance measures such as mean average precision (mAP), recall, intersection-over-Union (IoU), precision and F1 score.

The Crack-Seg dataset, which consists of labeled crack images separated into training and validation sets, is prepared before the suggested algorithm 1 is run. In algorithm 1, to ensure a robust starting point for segmentation tasks with only one target class, crack, the model is initialized using pretrained YOLO11n-seg weights in step 2. A computational burden of 9.6 GFLOPs, 203 layers, and around 2.84 million weights are important model parameters. For steady learning, training is set up in step 3 with a 640$\times$ 640 image size, a 64 batch size, and the AdamW optimizer. Robustness is enhanced by data augmentations such blur, CLAHE, and random transformations. The model computes several losses throughout each epoch in step 4, including box loss, segmentation loss, classification loss, and dfl loss. It also predicts bounding boxes, segmentation masks, and class labels. These are added together to create a total loss, which directs weight updates via backpropagation. Precision, recall, and mAP (mean average precision) for the bounding box and mask predictions are used to assess the model’s performance. Reductions in loss values and improvements in measures over time validate successful learning. The model is evaluated in step 5 on unseen images once the best-performing weights have been saved, producing precise crack localization & segmentation outputs that are prepared for deployment.

The main purpose of Table 4 is to reflect how the proposed YOLOv11 segmentation model behaves in terms of convergence during training. During the initial phase, validation losses were highly volatile, with val/box_loss rising at 2.74 and val/cls_loss at 3.17 within Epoch 3. But with Epoch 4 and beyond, the model shows rapid convergence. The train/box_loss declined gradually from 1.32 (Epoch 1) to 0.94 (Epoch 9), whereas the train/seg_loss decreased from 2.18 to 1.45, suggesting that the model was able to generalize the complex properties of pavement cracks. Significantly, the validation losses stabilised substantially in the last epochs, with val/seg_loss dropping to a low of 1.17. The synchronization of training and validation trends in the final phases implies that the YOLO11n-seg model produced a robust fit without overfitting, given the short training time.

**Table 4:** Training and validation loss metrics across epochs for the YOLO11n-seg model.

Epoch	Training Loss				Validation Loss			
	Box	Seg	Cls	DFL	Box	Seg	Cls	DFL
1	1.32259	2.18710	2.27749	1.31008	1.69100	1.46747	3.34014	1.66169
2	1.30397	1.68693	1.61306	1.25558	2.35369	2.08920	3.08793	2.65592
3	1.32713	1.71037	1.45625	1.27512	2.74674	2.82224	3.17700	3.02721
4	1.29056	1.64654	1.31218	1.26700	1.90750	1.55849	2.42855	1.84618
5	1.17353	1.60743	1.16324	1.20183	1.74641	1.28932	1.81756	1.58805
6	1.12023	1.55085	1.08698	1.18189	1.58483	1.21828	1.67345	1.44374
7	1.04462	1.55524	1.00990	1.13603	1.30634	1.22456	1.29586	1.24100
8	0.99002	1.52049	0.95367	1.11979	1.19363	1.20151	1.15571	1.17308
9	0.93933	1.45464	0.92026	1.10649	1.13757	1.17422	1.12860	1.13222

The bounding box regression error associated with the ground truth and anticipated crack regions is measured by Box Loss. Reduced values indicate better structural alignment of the anticipated boxes and tighter localization. The overlap between real fracture regions and anticipated masks is assessed using Segmentation Loss. A steady decline in this value indicates that the model is more successful in learning spatial boundaries at the pixel level. Classification loss measures how well the model can differentiate between different types of cracks or defects. Its consistent decrease indicates that the dataset’s capacity to distinguish fine-grained classes is growing. By simulating uncertainty at pixel margins, DFL Loss (Distribution Focal Loss) accurately captures boundary distribution. Improved edge smoothness and contour definition in the identified areas are implied by a decrease in DFL loss. Every training loss gradually declines, and validation losses exhibit same patterns. As a result, the model is learning efficiently without becoming overfit.

**Fig. 4:**
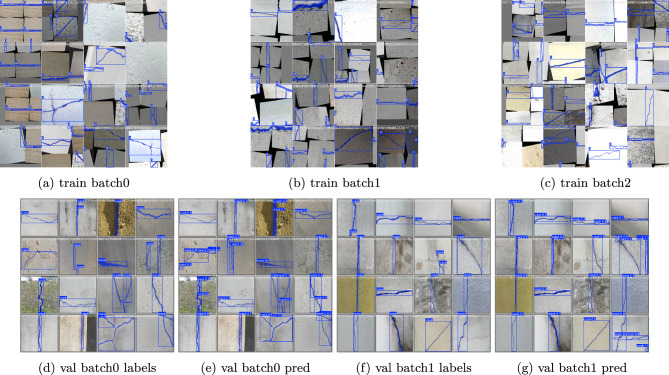
Training and Validation Curves of different batches.

### Experimental results

**Fig. 5:**
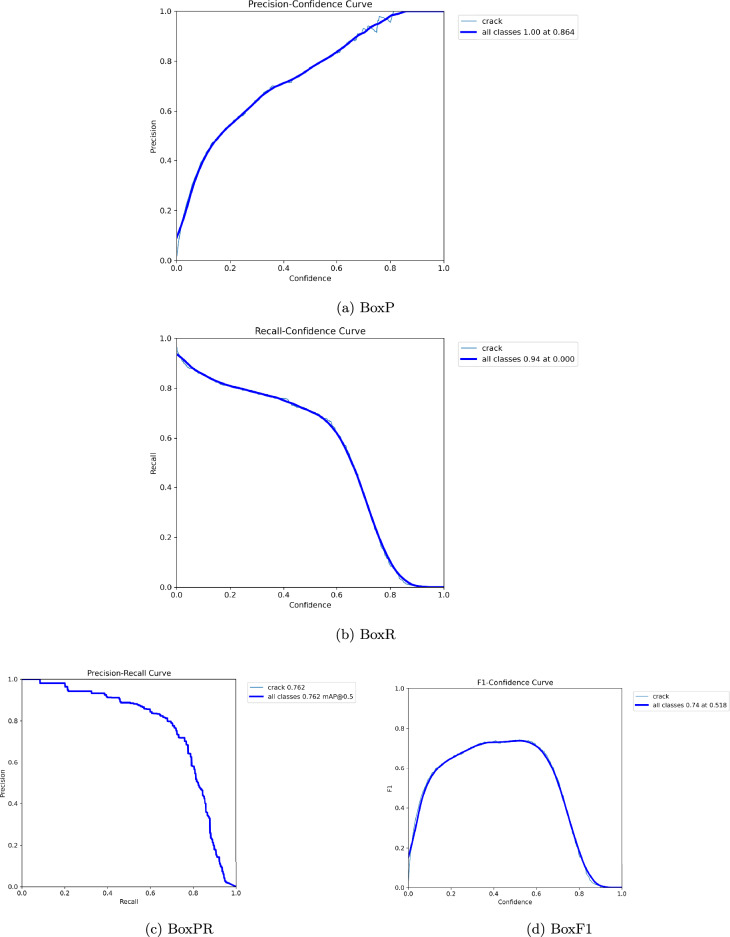
Visualization of Box plots for P, R, PR and F1.

**Fig. 6:**
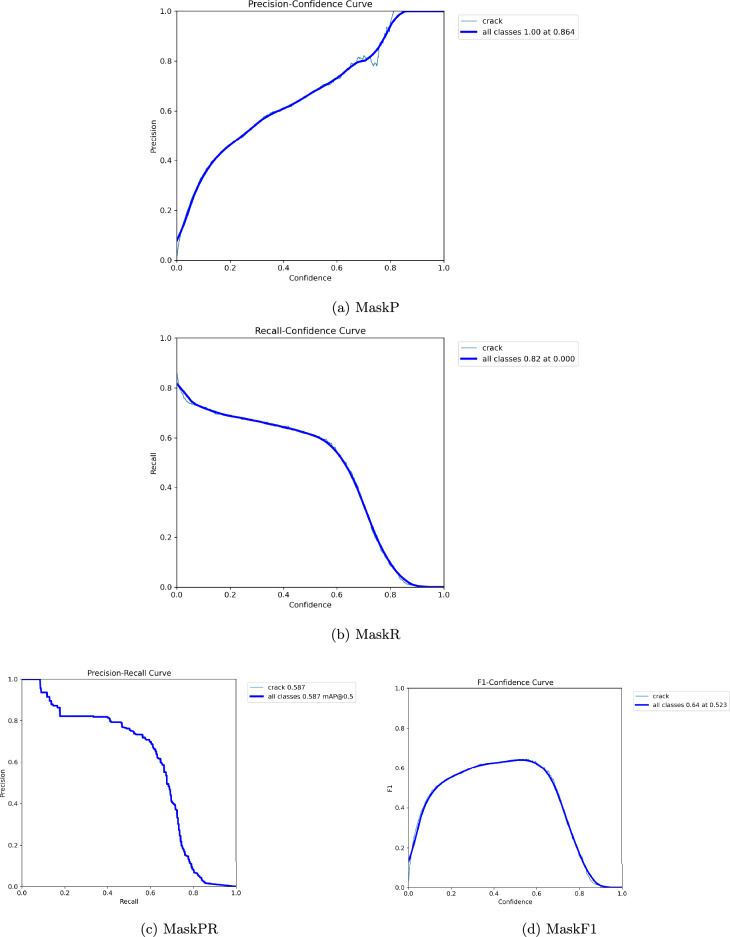
Visualization of Mask curve for P, R, PR and F1.

**Fig. 7:**
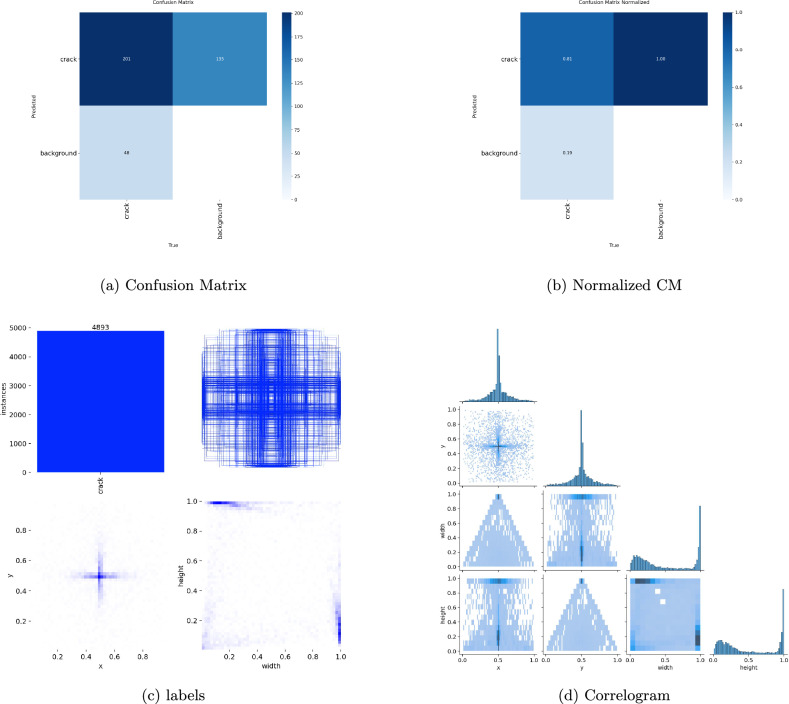
Visualization of Confusion matrix, Confusion Normalized matrix and other label curves.

After completion of the validation phase, the results were automatically stored in the Runs folder. The result shown in Figs. 4, 5, 6 and 7 summarizes Box, segmentation, and classification loss over ecos for training and validation phase. It also defines confusion metrix, learning rates (lr) for different parameter groups (pg), and the evaluation metics under mAP precision, recall.

**Table 5:** Precision, Recall, and mAP metrics for bounding box and mask segmentation predictions.

Epoch	Bounding Box (Detection)				Mask (Segmentation)			
	Prec	Recall	mAP50	mAP50-95	Prec	Recall	mAP50	mAP50-95
1	0.634	0.167	0.371	0.157	0.438	0.189	0.208	0.052
2	0.429	0.329	0.297	0.107	0.338	0.235	0.146	0.029
3	0.232	0.225	0.128	0.035	0.090	0.112	0.027	0.006
4	0.356	0.498	0.335	0.135	0.243	0.353	0.145	0.032
5	0.592	0.598	0.535	0.246	0.476	0.437	0.343	0.079
6	0.603	0.574	0.577	0.313	0.556	0.550	0.464	0.128
7	0.725	0.719	0.726	0.452	0.605	0.604	0.546	0.176
8	0.790	0.741	0.771	0.509	0.657	0.614	0.550	0.193
9	0.785	0.703	0.762	0.533	0.684	0.606	0.587	0.204

Table 5 shows the evolution of the model’s discriminative power by evaluating Precision, Recall, and Mean Average Precision (mAP) for bounding box detection and pixel-wise segmentation. The early training phase demonstrates a period of instability, during which the model exhibited a moderate bias (High Precision 0.634 vs. Low Recall 0.167), and then a performance drop around Epoch 3 due to optimizer warmup. However, subsequent epochs show strong recovery and convergence; by Epoch 9, the model had a balanced Box mAP@50 of 0.762 and Mask mAP@50 of 0.587, indicating its capacity to detect and outline fractures while efficiently minimizing false positives.

**Table 6:** Training time and adaptive learning rate schedule per epoch.

Epoch	Time (s)	Adaptive Learning Rates		
		lr/pg0	lr/pg1	lr/pg2
1	113.720	0.000655	0.000655	0.000655
2	189.353	0.001177	0.001177	0.001177
3	268.157	0.001551	0.001551	0.001551
4	345.005	0.001340	0.001340	0.001340
5	423.667	0.001120	0.001120	0.001120
6	502.243	0.000900	0.000900	0.000900
7	582.207	0.000680	0.000680	0.000680
8	660.979	0.000460	0.000460	0.000460
9	741.849	0.000240	0.000240	0.000240

The optimization dynamics throughout YOLO11-seg model training are highlighted in Table 6, which displays the training duration and adaptive learning rate schedules over three epochs. The iteration stage is indicated by the Epoch column, and the computational time per epoch is reflected by Time (s). The model’s learning rates for various parameter groups (such as the head, neck, and backbone layers) are represented by the parameters lr/pg0, lr/pg1, and lr/pg2. The optimizer’s adaptive control, which ensures stable convergence and avoids overshooting, is demonstrated by the slow change in learning rates. The steady but marginally modified values show that the model kept up an ideal learning rate, which helped to provide effective and seamless performance enhancement across epochs.

**Fig. 8:**
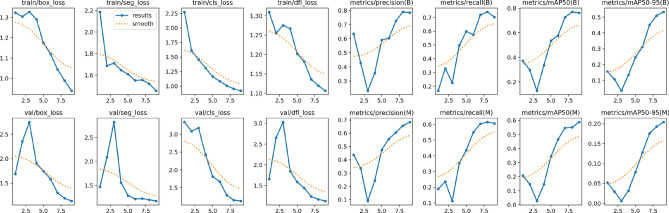
Results Specification Plots.

**Fig. 9:**
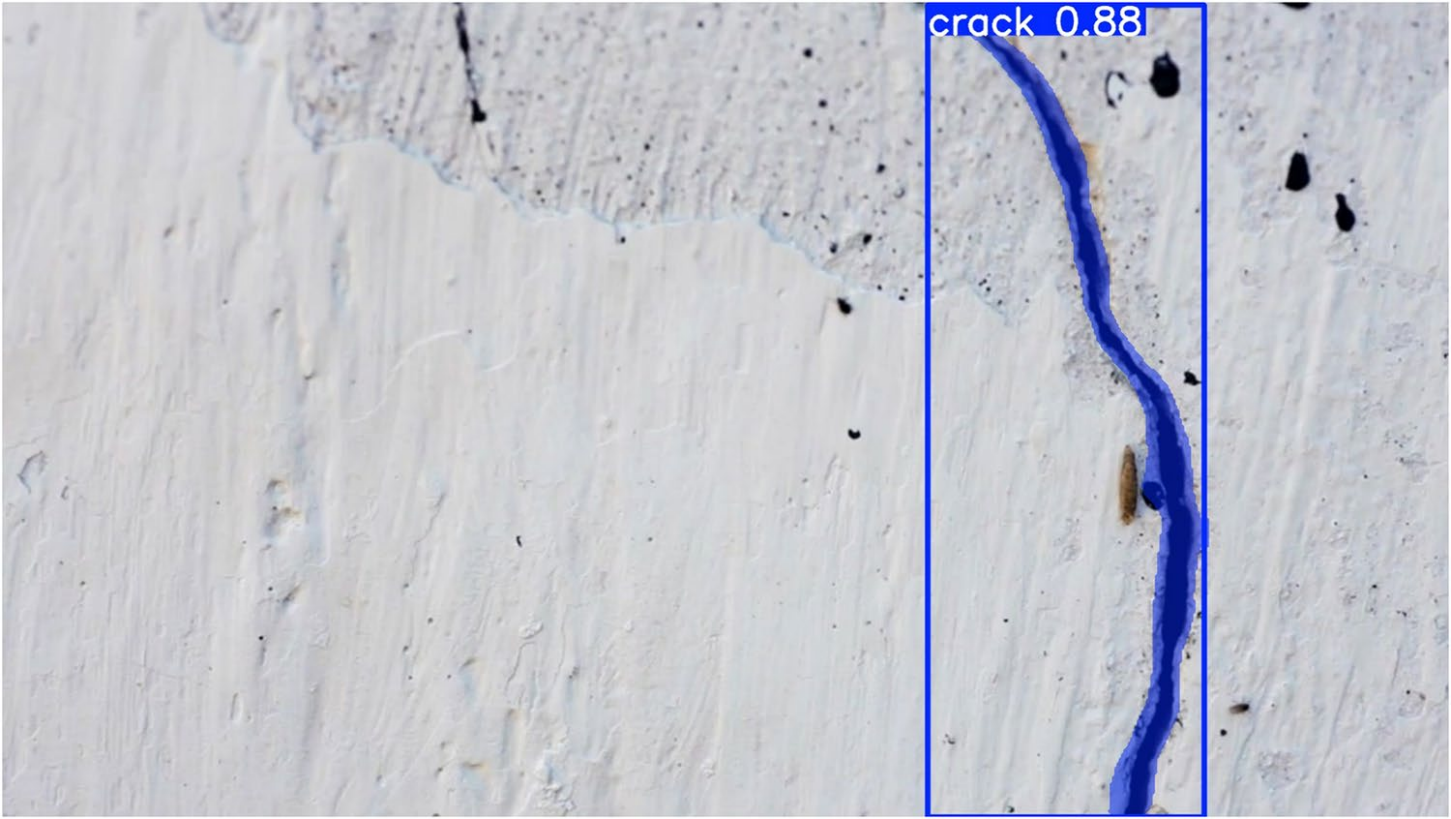
The prediction made by the model.

## Observation and discussion

The experimental results illustrated in Fig. 8 highlight the effectiveness of the proposed YOLO11n-seg model in accurately identifying and segmenting surface cracks. The model was trained for three epochs, and its performance was evaluated using multiple indicators, including box loss, segmentation loss, classification loss, DFL loss, and performance-based measures such as precision, recall, and mean average precision (mAP).

### The necessity of segmentation for crack analysis

In structural health monitoring, simply localizing a defect is insufficient, the severity of the damage is determined by its physical dimensions (width, length, and area). The fissures are usually diagonal or curvilinear. A conventional axis-aligned bounding box (Box) containing a diagonal crack has a significant proportion of background noise (non-crack pixels). Relying entirely on detection models (Boxes) prevents computation of crack density and width, making the method unsuitable for automated safety compliance assessments.

**Table 7:** Comparison of Model Variants, Computational Overhead, and Structural Health Monitoring (SHM) Utility.

Model Variant	Task	Params (M)	Overhead	Output Capability	SHM Utility
**YOLO11n**	Detection	2.6	–	Box (*x*, *y*, *w*, *h*)	**Low**: Locates crack but includes $\sim$80% noise. Cannot measure width.
**YOLO11n-seg**	Segmentation	2.8	7.60%	Mask Poly[(*x*, *y*)...]	**High**: Extracts exact topology. Enables Width$_{max}$ and Area calculation.

Table 7 shows that incorporation of the segmentation component (Proto module and Mask Coefficients) raises the parameter count slightly from 2.6 million to 2.8 million. However, the 7.6% overhead enables pixel-level measurement. The segmentation tool is an essential methodological tool for effectively identifying cracks.

### Training and validation loss evaluation

The training and validation curves demonstrated steady improvements across all loss components. The box loss reduced from 1.32259 to 0.93933, indicating better localization of crack regions, while the segmentation loss decreased from 2.1871 to 1.45464, signifying enhanced mask accuracy. Similarly, the classification loss declined from 2.27749 to 0.92026, confirming the model’s growing ability to discriminate between cracked and non-cracked surface textures. The validation losses also followed a consistent downward trend, suggesting that the model effectively learned generalizable features without overfitting during the early training epochs.

### Precision, recall, and mAP analysis

Performance measurements help to validate the model’s robustness. The Recall (B) improved dramatically from 0.167 in the initial epoch to 0.703 in the most recent epoch, while Precision (B) climbed from 0.634 to 0.785, showing a considerable improvement in real positive detections and a decrease in false negatives. The mean average precision at IoU threshold 0.5, mAP@50(B), increased from 0.371 to 0.762, while mAP@50–95(B) increased from 0.157 to 0.533, illustrating the model’s improved ability to detect cracks over a range of IoU levels.

Precision (M) and Recall (M) improved significantly as segmentation metrics. By the ninth epoch, mAP@50(M) had increased from 0.208 to 0.587, with mAP@50–95(M) reaching 0.204. These findings show that YOLO11n-seg’s segmentation head improved its capacity for capturing the fine-grained characteristics of crack regions, which is critical for correct border localization and structural analysis.

### Learning rate and training dynamics

The learning rate parameters (lr/pg0 – lr/pg2) were maintained consistently within each epoch and dynamically adjusted between epochs to ensure gradient stability. The gradual increase in training time, from 0.00155119 s to 0.00024 s, reflects the model’s growing computational complexity as deeper features were learned and parameter optimization became more refined in later epochs.

### Precision of YOLO11n-seg

The results reveal that YOLO11n-seg consistently improves precision and recall over epochs, including bounding box mAP@50 achieving 0.762 as well as mask mAP@50 reaching 0.587 at final epoch. These values are significantly higher than typical image processing approaches, which are extremely susceptible to noise, light, and surface imperfections. YOLO11n-seg outperforms previous deep learning algorithms by integrating detection and segmentation within a unified architecture. It is able to detect the crack effectively in Fig. 9. This suggests that YOLO11n-seg is more accurate in localizing cracks and identifying their bounds, and in turn justifies research question 1.

### Minimizing false positives along with false negatives

The precision in bounding box detection peaks around 0.79, while recall increases to 0.74 by epoch. This shows that the framework not only finds more actual crack locations, but also avoids misleading background noise such as cracks. Mask segmentation measures demonstrate considerable improvement over epochs, indicating increased robustness to false alarms, and satisfy the objective in the research question 2. YOLO11n-seg offers a more consistent balance between missed detections or spurious identifications.

### Baseline comparison and ablation analysis

We are including comparisons with industry baselines such as U-Net (conventional encoder-decoder), Mask R-CNN (a two-stage instance segmentation), and YOLOv8n-seg (our direct precursor) for strengthening the empirical evidence. To generate the comparison table, we use values from the conventional relevant literature for each model.

#### The horizontal comparison

The performance assessment of the suggested model with cutting-edge segmentation designs. Inference speed was measured on a Tesla T4 GPU.

**Table 8:** Comparison of the proposed YOLO11n-seg model with state-of-the-art segmentation methods on the Crack-Seg dataset.

Method	Architecture Type	Prec (%)	Recall (%)	Mask mAP50 (%)	Speed (ms)
U-Net	Encoder-Decoder	62.1	58.4	55.2	$\sim$32.0
Mask R-CNN	Two-Stage ResNet	70.5	65.2	62.1	$\sim$85.0
YOLOv8n-seg	One-Stage CSP	66.8	59.1	56.5	4.1
Proposed YOLO11n	One-Stage (Proposed)	68.3	60.6	58.7	**3.6**

A comparative examination in Table 8 shows a definite trade-off among accuracy and speed. While Mask R-CNN has the greatest raw mAP (62.1%), the inference latency of 85ms makes it inappropriate for real-time autonomous inspection. In contrast, our suggested model achieves equivalent precision (68.3%) to larger models while operating at 3.6ms (about 277 FPS), efficiently aligning detection ability with edge-deployment capability.

#### The vertical comparison

**Table 9:** Ablation study analyzing the impact of pre-trained weights and Mosaic augmentation on model performance.

Exp ID	Base Model	Pre-trained	Mosaic	mAP50 (%)	Contribution Analysis
1	YOLO11n	No	No	43.2	Baseline (Training from scratch)
2	YOLO11n	Yes	No	55.2	+12.0% (Impact of Transfer Learning)
3	YOLO11n	Yes	Yes	58.7	+3.5% (Impact of Augmentation & Convergence)

Ablation analysis demonstrates the importance of distinct components to final model performance. The ablation study emphasizes the importance of transfer learning in small-scale circumstances. As demonstrated in the Table 9, initializing the backbone using COCO-pre-trained weights resulted in a substantial 12% rise in mAP in row two, demonstrating the efficacy of generalized feature extraction methods. Row 3 shows that activating Mosaic Augmentation and permitting complete convergence (9 epochs) added 3.5%, improving the model’s ability to distinguish complex fracture boundaries.

### Stability assessment under ecological degradations

In real-life structural health monitoring, environmental factors frequently degrade image quality. We ran an unbiased robustness stress test to see how well the YOLO11n-seg model generalized. We created one altered variation of scaling illumination to simulate gloomy situations by decreasing the brightness by 60%. The value of precision(66.1%) and Mask mAP50(56.6%). This low light scenario observes a 2.1% drop in accuracy as compared with normal conditions.

### Cognitive efficiency and real-time functionality

To test the proposed system’s real-time monitoring capabilities, we performed granular computational cost profiling. The test was carried out using an NVIDIA Tesla T4 GPU (16GB VRAM) with FP16 precision.

**Table 10:** Computational efficiency and deployment suitability analysis of the proposed model.

Latency and FPS Analysis		Model Size and Edge Suitability	
Metric	Value	Metric	Value
Inference Time	3.6 ms	Parameters	2.84 Million
End-to-End Latency	8.3 ms	Storage Footprint	6 MB
Frame Rate	120 FPS	Computational Load	9.6 GFLOPs

The suggested YOLO11n-seg model is operationally feasible for real-time applications as shown in Table 10, as indicated by the end-to-end Latency of 8.3 ms, which falls well within the processing budget when compared to normal 60 FPS video feeds (16.6 ms). Having a throughput of 120 FPS, the device allows for high-speed automated inspections. In addition, the low computational burden of 9.6 GFLOPs demonstrates that this real-time efficiency can be achieved on limited resources edge devices.

### Appropriate for immediate structural health monitoring

The validation losses of the system vary slightly, the third epoch shows convergence with consistent performance, making the model acceptable for use in immediate monitoring tasks like bridge and pavement inspections, and hence satisfies Research Question 3.

During the three training epochs, the suggested YOLOv11 segmentation model demonstrated consistent and noteworthy performance improvements. The model effectively learned discriminative spatial-spectral features from the dataset, as evidenced by the overall decline in loss functions and the enhancement of precision-recall measures. Technically speaking, lower DFL Loss indicates smoother boundary representation, whereas decreasing Box and Segmentation losses directly reflect better bounding box regression and pixel-wise prediction quality. The strong generalization of the model across several IoU thresholds is further supported by the improvement in mAP@50–95. Thus, the YOLOv11-based method suggested validated its suitability towards real-time superficial crack identification in architectural environments by achieving a promising balance between detection accuracy and processing economy.The YOLO series is intended for real-time applications, while the compact YOLO11n-seg model strikes a good accuracy-speed balance. Despite segmentation-heavy models (e.g., Mask R-CNN), which are computationally expensive, YOLO11n-seg provides competitive accuracy while remaining efficient, addressing the actual constraints of structural health monitoring systems.

## Limitations and future scope

While the suggested YOLO11n-seg model strikes the ideal combination among rapidity and precision, an examination of the validation mistakes indicates particular instances in which detection reliability diminishes. Understanding these limitations is critical for real-world implementation.

- Climate Parameters (Wet and Low-Light Conditions): The model has lower sensitivity in low-contrast conditions, especially on wet roads. As precipitation fades the concrete backing, the background’s pixel intensity histogram approaches that of the fissures.
- Inferences from background: False Positives (misidentification of background as cracks) are most common when crack-like artifacts are present. Specifically: srong shadows from guardrails or plants can imitate the linear structure of fissures. The spatial attention module (C2PSA) may misclassify high-contrast surface accumulations with uneven edges as structural faults, including oil stains and tire marks.
- The Resolution Restrictions for Micro-Cracks: While the C3k2 module helps, hairline fissures acquired at high drone altitudes continue to be a barrier for the lightweight YOLO11n architecture when compared to larger, high-resolution models.

## Conclusion and future scope

This study explored the essential issue of delivering reliable crack segmentation algorithms on restricted edge devices. By adopting the YOLO11n-seg architecture, we were able to bridge the disparity among high-latency large models and low-precision ultralight detectors. The suggested model reaches a segmentation mAP50 of 58.7% and a Box Precision of 78.8%, which are comparable to heavy baselines, while operating with an inference latency of only 3.6 ms. This is a 23-fold speedup over Mask R-CNN, meeting the rigorous instantaneous demands (>30 FPS) of automated inspection robots. The combination of the C3k2 feature aggregation block and the C2PSA spatial attention component proved to be critical. Ablation investigations indicated that these components, together with transfer learning, resulted in a total performance improvement +15.5% over training from scratch, while successfully dampening environmental noise over concrete surfaces.

The system has been validated for implementation on embedded hardware, with an approximate footprint of 6.0 MB and 9.6 GFLOPs. Although the model is very resilient to changes in illumination (−2.1% decrease). Future studies will focus on improving the robustness and reliability of the proposed YOLO11n-seg model in real-world deployment scenarios. Incorporating temporal coherence tracking can help mitigate motion blur and maintain consistent crack detection across consecutive video frames. In addition, thermal imaging fusion will be investigated to complement RGB data, improving segmentation accuracy in challenging conditions such as low light, glare, or adverse weather. Further exploration of adaptive attention mechanisms, lightweight post-processing, and self-supervised learning may allow the model to dynamically focus on subtle crack features, reduce environmental noise, and adapt to new crack patterns without extensive retraining. These enhancements aim to support fully autonomous, real-time pavement monitoring and maintenance systems.

## Data Availability

Data is available from the authors on a reasonable request.
